# Trajectory Design for Multi-UAV-Aided Wireless Power Transfer toward Future Wireless Systems

**DOI:** 10.3390/s22186859

**Published:** 2022-09-10

**Authors:** Jun Mu, Zhaojie Sun

**Affiliations:** 1 Space Star Technology Co., Ltd., Beijing 100086, China; 2 The Key Laboratory of Science and Technology on Communications, University of Electronic Science and Technology of China, Chengdu 611731, China

**Keywords:** wireless power transfer (WPT), UAV trajectory design, energy optimization

## Abstract

In this paper, we investigate an unmanned aerial vehicle (UAV)-assisted wireless power transfer (WPT) system, in which a set of UAV-mounted mobile energy transmitters (ETs) are dispatched to broadcast wireless energy to an energy receiver (ER) on the ground. In particular, we aim to maximize the amount of energy transferred to the ER during a finite UAV’s flight period, subject to the UAV’s maximum speed and collision avoidance constraints. First, the basic one/two-UAV scenarios are researched in detail, which show that UAVs should hover at fixed locations during the whole charging period. Specifically, the Lagrange multiplier method is employed to solve the proposed optimization problem for the case of two UAV situation. Specifically, the general conclusions based on the theoretical analysis of one/two-UAV scenarios are drawn contribute to deducing the trajectory design of UAVs when the number of UAVs increases from three to seven. The obtained trajectory solution implies that UAVs should be evenly distributed on the circumference with point (0,0,H) as the center and UAVs’ safe distance as the radius. Finally, numerical results are provided to validate the trajectory design algorithm for the multiple UAVs-enabled single-user WPT system.

## 1. Introduction

### 1.1. Background

The limited battery life of wireless devices has often been a key consideration in the performance of wireless communication, since manual battery replacement/recharging would cause frequent disruptions. The recent advances in radio frequency (RF)-enabled wireless power transfer (WPT) technology [1,2] offer a promising solution toward future wireless communications [3,4,5], in which wireless devices use collected RF energy to transmit/decode information to/from other devices. As a solution, RF-enabled WPT has emerged as an attractive technique to provide convenient and cost-effective energy supplies to lower-power wireless networks, such as the upcoming Internet-of-Things (IoT) systems [6,7] consisting of millions of sensors [8,9,10]. In addition, the WPT technology employed in communication is anticipated to improve quality-of-service (QoS), with higher throughput and robustness compared to conventional battery-powered counterparts.

The conventional WPT systems with fixed energy transmitters (ETs) face several challenges [11,12] and the performance of practical WPT systems is fundamentally restricted by the low end-to-end power efficiency on account of the serious propagation loss of RF signals over long distances. Therefore, with the purpose of constituting ubiquitous wireless energy access points (APs) to charge tremendous low-power energy receivers (ERs) distributed in a large area, it is necessary to design the deployment of ETs in a super-dense manner. However, this will bring a significantly higher cost, resulting in hindering a large-scale implementation of the future WPT systems. Recently, motivated by the advantages of UAV-assisted wireless communications, UAV-enabled WPT has become a promising solution to the problems faced by conventional WPT system.

Due to the flexibility and cost-efficiency characteristics, UAVs have been employed in wide range of applications, such as military operations, cargo delivery, aerial cameras, and communication platforms, etc. [13,14,15,16,17,18]. In particular, low-altitude UAVs equipped with miniaturized communication transceivers can be utilized as aerial mobile base stations (BSs) to expand the communication coverage for ground users [19,20,21,22,23] or relays to enhance the system throughput [24,25,26,27,28]. The link distances from the UAV to its served ground users can be effectively shortened by jointly optimizing the UAV’s trajectory and communication resource allocation, and thus significant enhancement of system throughput can be achieved [29,30,31,32].

### 1.2. Related Work and Motivation

Nowadays, UAV-enabled WPT has been investigated in [11,33,34,35,36,37,38], in which UAVs are applied as mobile ETs to supply low-power ERs distributed in the given area. Specifically, considering the UAV-enabled WPT systems where the UAV is flying at a fixed height, the authors maximized the energy transfer performance by optimizing the one-dimensional (1D) or two-dimensional (2D) UAV trajectory under the constraints of maximum UAV speed [11,33]. To be more specific, the authors in [33] characterized the Pareto boundary of the achievable energy region through optimizing the 1D UAV trajectory in the case of dual users. A more general case with the 2D WPT system and multiple users was then extended in [11], where the heuristic successive hover-and-fly (SHF) trajectory and the successive convex approximation (SCA) technique were performed to maximize the minimum received energy among all users. Different from the works [11,33,34,35,36] that only provided heuristic and locally optimal solutions, the authors in [39] presented a globally optimal 1D UAV trajectory design for solving the considered min-energy maximization problem for the first time.

In addition, numerous literatures adopted the RF-based wireless energy transfer (WET) methods applied to UAV-aided wireless powered communication network (WPCN), where UAVs fly towards a set of ground users periodically to transmit information in the downlink and then receive uplink information [40,41,42,43]. A single user case was considered in [40], where the UAV trajectory was fixed to a line segment, while the UAV-aided WPCN where UAVs with arbitrary trajectories serve multiple energy-constrained users was investigated in [41]. Furthermore, the trajectories of the UAVs and wireless resource allocation were jointly optimized to maximize the minimum throughput of the users [41,42]. To provide the required quality of services (QoS) to massive ground users distributed in a large area, the cooperative UAV trajectory design and wireless resource allocation problem was investigated in [43,44] for multi-UAV-enabled WPC systems under a basic setup with two UAVs serving two users.

Different from the previous studies, the previous works mainly focused on the scenarios where one or two UAVs serve one or more ground users. In practice, it is necessary to utilize multiple UAVs to cooperatively serve one or many ground users in a large area in order to improve the WPT’s energy performance. This thus motivated our investigation in this work. Please allow us to respond to your questions as follows.

### 1.3. Contributions

In this paper, we consider the UAV-enabled WPT system with multiple UAVs serving one terrestrial ER. Under such a setup, we maximize the sum received energy of the ER over a particular UAV mission period by optimizing the UAVs’ trajectories subject to the UAV’s maximum flying speed and collision avoidance constraints. The main contributions of this work are as follows:

- Different from the existing works, we consider the UAV-enabled WPT system focusing on the cooperation of multiple UAVs to provide wireless energy to one ER on the ground. Under the constraints of UAV’s maximum speed and collision avoidance, we optimize the trajectories of UAVs to maximize the sum received energy of the ER.
- With the purpose of obtaining general conclusions, in Section 3, we conduct specific analysis about the cases where the number of UAVs is 1 and 2, respectively. In particular, we adopt the Lagrange multiplier method to solve the proposed optimization problem for a two-UAV-enabled WPT system.
- Based on the analysis of the one/two-UAV-enabled WPT system, the general conclusions are obtained in Section 4, which is beneficial to deduce the trajectories design of UAVs when the number of UAVs increases from three to seven. Furthermore, we also conduct a detailed analysis about the case where one UAV serves two ERs. Numerical results show that our presented trajectory design scheme is completely correct.

The remainder of this paper is organized as follows. The detailed system model and general sum-energy maximization problem are introduced in Section 2. In Section 3, we deeply investigate one/two-UAV-aided WPT systems. Building on the characterization, we further propose a general trajectory design scheme for when the number of UAVs increases from three to seven in Section 4. Finally, we present numerical results which validate the effectiveness in Section 5, and we conclude in Section 6.

## 2. System Model

**Figure 1:**
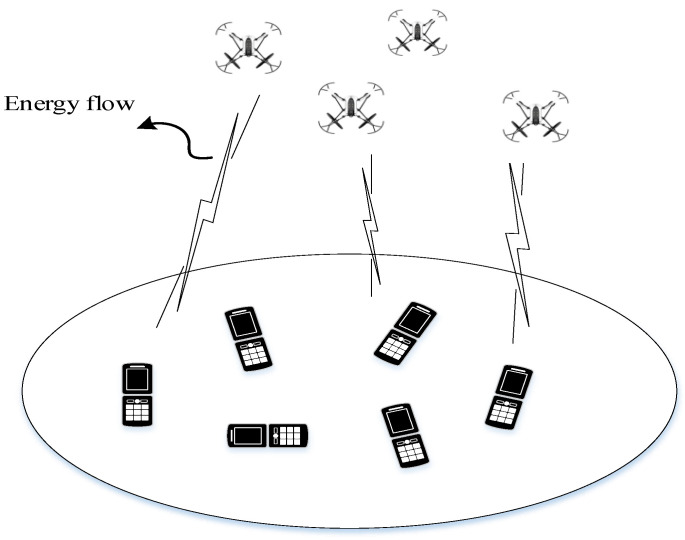
The system model of UAV-aided WPT.

As portrayed in Figure 1, consider a UAV-assisted WPT scenario, where *M* UAVs are dispatched to broadcast wireless energy to charge *K* ERs distributed on the ground.

Let $M=\left\{1,\cdots ,M\right\}$ denote the set of UAVs and $K=\left\{1,\cdots ,K\right\}$ denote the set of ERs. Suppose that each ER k∈K has a fixed location $\left(x_{k},y_{k},0\right)$ in a three-dimensional (3D) Euclidean coordinate, which is assumed to be known a priori by the UAVs to facilitate their trajectory designs. We focus on a finite charging period $T=\left[0,T\right]$ with duration *T* in second (s), in which all UAVs are assumed to fly horizontally at a fixed altitude H>0 in meters (m). Accordingly, at each time instant t∈T, the time-varying location of UAV m∈M is denoted as $\left(X_{m}\left(t\right),Y_{m}\left(t\right),H\right)$. Furthermore, let *V* in meters/second (m/s) denote the maximum possible speed of the UAV and Δ denote the minimum inter-UAV distance to avoid collision. Therefore, the maximum flying speed and collision avoidance constraints at each time instant can be expressed as:

(1) $$\sqrt{{\left(\frac{dX_{m}\left(t\right)}{dt}\right)}^{2}+{\left(\frac{dY_{m}\left(t\right)}{dt}\right)}^{2}}\leq V,t\in \left[0,T\right],m\in M,$$

(2) $$\sqrt{{\left(X_{m}\left(t\right)-X_{m^{\prime }}\left(t\right)\right)}^{2}+{\left(Y_{m}\left(t\right)-Y_{m^{\prime }}\left(t\right)\right)}^{2}}\geq \Delta ,t\in \left[0,T\right],m,m^{\prime }\in M.$$

Since the wireless channels between the UAVs and each ER are normally dominated by line-of-sight (LoS) links, the free-space path loss model is similar as [9,10,15,20], which is, in general, a practical assumption. Accordingly, the channel power gain from UAV m∈M to ER k∈K at time t∈T is modeled as:

(3) $$h_{k,m}\left(t\right)=\frac{\beta_{0}}{d_{k,m}^{2}\left(t\right)},$$

where $d_{k,m}\left(t\right)$ is the distance between UAV m∈M and ER k∈K, and it can be specifically formulated as:

(4) $$d_{k,m}\left(t\right)=\sqrt{{\left(X_{m}\left(t\right)-x_{k}\right)}^{2}+{\left(Y_{m}\left(t\right)-y_{k}\right)}^{2}+H^{2}}.$$

Additionally, $\beta_{0}$ represents the channel power gain at a reference distance of $d_{0}=1$ m. Assuming that each UAV transmits with fixed transmit power *P*, the collected RF power by ground ER *k* at time t∈T is thus given by:

(5) $$\begin{array}{c} Q_{k}\left({\left\{X_{m}\left(t\right),Y_{m}\left(t\right)\right\}}_{m=1}^{M}\right)=\sum_{m=1}^{M}\eta h_{k,m}\left(t\right)P\\ \;\;\;\;\;\;\;\;\;\;\;\;\;\;\;\;\;\;\;\;\;\;\;\;\;\;\;\;\;\;\;\;\;\;\;\;\;\;\;\;\;\;\;\;\;\;\;\;=\sum_{m=1}^{M}\frac{\eta \beta_{0}P}{{\left(X_{m}\left(t\right)-x_{k}\right)}^{2}+{\left(Y_{m}\left(t\right)-y_{k}\right)}^{2}+H^{2}}, \end{array}$$

where 0<η≤1 represents the RF-to-direct current (DC) energy conversion efficiency at the rectifier of each ER. Accordingly, the available energy harvested by ER k∈K over the given charging period is a function of the UAV’s trajectory $\left\{X_{m}\left(t\right),Y_{m}\left(t\right)\right\}$, which can be expressed by:

(6) $$E_{k}\left({\left\{X_{m}\left(t\right),Y_{m}\left(t\right)\right\}}_{m=1}^{M}\right)=\int_{0}^{T}Q_{k}\left({\left\{X_{m}\left(t\right),Y_{m}\left(t\right)\right\}}_{m=1}^{M}\right)dt.$$

In this paper, our objective is to maximize the sum received energy among all ERs over the charging period by optimizing the UAVs’ trajectory ${\left\{X_{m}\left(t\right),Y_{m}\left(t\right)\right\}}_{m=1}^{M}$ with the UAV’s maximum speed constraints and collision avoidance constraints. As a result, the problem can be mathematically formulated as:

(7) $$\begin{array}{c} \max_{{\left\{X_{m}\left(t\right),Y_{m}\left(t\right)\right\}}_{m=1}^{M}}\;\;\;\underset{k}{sum}E_{k}\left({\left\{X_{m}\left(t\right),Y_{m}\left(t\right)\right\}}_{m=1}^{M}\right)\\ \;\;\;\;\;\;\;\;\;\;\;\;s.t.\;\;\;\;\;\;\;\;\;\;\;\;\;\;\;\;\left(1\right)\left(2\right) \end{array}$$

The cooperation among UAVs is particularly considered in this paper, for the convenience of analysis, we study the multi-UAV-enabled single-user WPT. To draw more insights, we firstly study the one/two-UAV-enabled WPT system in a careful way in Section 3. Next, Section 4 presents a general trajectory design scheme for when the number of UAVs increases from three to seven based on the conclusions drawn in Section 3.

## 3. One/Two-UAV-Enabled WPT System

### 3.1. Special Case with M = 1 UAV

In this subsection, we consider Equation (7) for the special case with M=1, K=1. Without loss of generality, suppose the coordinates of terminal ER are $\left(0,0,0\right)$ and the position of UAV is denoted as $\left(x\left(t\right),y\left(t\right),H\right)$. It is obvious that the collision avoidance constraint is redundant. Consequently, the optimization problem in (7) can be reduced to:

(8) $$\begin{array}{c} \max_{\left(x\left(t\right),y\left(t\right)\right)}\;\;\;\int_{0}^{T}\frac{\eta \beta_{0}P}{x^{2}\left(t\right)+y^{2}\left(t\right)+H^{2}}dt\\ \;\;\;\;\;\;s.t.\;\;\;\;\;\;\;\;\;\left(1\right) \end{array}$$

In order to solve the optimization problem in (8), we first consider a relaxed problem without constraints, which can be expressed as:

(9) $$\max_{\left(x\left(t\right),y\left(t\right)\right)}\;\;\;\int_{0}^{T}\frac{\eta \beta_{0}P}{x^{2}\left(t\right)+y^{2}\left(t\right)+H^{2}}dt.$$

By introducing $f\left(t\right)=\frac{\eta \beta_{0}P}{x^{2}\left(t\right)+y^{2}\left(t\right)+H^{2}}$ and converting the integral to the sum, Formula (9) can be rewritten as:

(10) $$\max_{\left(x\left(\frac{T}{N}i\right),y\left(\frac{T}{N}i\right)\right)}\;\;\;\lim_{N\to \infty }\frac{T}{N}\sum_{i=1}^{N}f\left(\frac{T}{N}i\right).$$

It is apparent that the limit operation can be moved up to the front, namely:

(11) $$\lim_{N\to \infty }\frac{T}{N}\max_{\left(x\left(\frac{T}{N}i\right),y\left(\frac{T}{N}i\right)\right)}\sum_{i=1}^{N}f\left(\frac{T}{N}i\right).$$

Additionally, the maximization operation in (11) can be decomposed into *N* sub-problems, i.e.,

(12) $$\max_{\left(x\left(\frac{T}{N}i\right),y\left(\frac{T}{N}i\right)\right)}f\left(\frac{T}{N}i\right),i=1,\cdots ,N.$$

By replacing $\frac{T}{N}i$ with *t*, the optimization problem can be converted to:

(13) $$\max_{\left(x\left(t\right),y\left(t\right)\right)}\;\;\;\frac{\eta \beta_{0}P}{x^{2}\left(t\right)+y^{2}\left(t\right)+H^{2}},\forall t\in \left[0,T\right].$$

Apparently, the optimal solution for (13) is not related to *t*, namely, $x\left(t\right)$ and $y\left(t\right)$ are fixed under the optimal state. Assume that $x\left(t\right)=x,\;\;\;y\left(t\right)=y$, and, according to (13), we have:

(14) $$\max_{\left(x,y\right)}\;\;\;\frac{\eta \beta_{0}P}{x^{2}+y^{2}+H^{2}}.$$

Obviously, $\left(x,y\right)=\left(0,0\right)$ is the optimal solution for problem (14). On the other hand, since the optimal value of problem (9) is the upper bound of the one for problem (8), and $\left(x\left(t\right),y\left(t\right)\right)=\left(0,0\right)$ satisfies the constraints in (8), it is concluded that the optimal solution of the problem (8) is $\left(x\left(t\right),y\left(t\right)\right)=\left(0,0\right)$.

In conclusion, when M=1,K=1, the UAV statically hovers at $\left(0,0,H\right)$, which is directly above the energy receiver. In addition, the total harvested energy of the ER is:

(15) $$E_{K=1,M=1}=\frac{\eta \beta_{0}PT}{H^{2}}.$$

### 3.2. Special Case with M = 2 UAV

The UAV-enabled WPT system for a basic setup with two UAVs serving one ER is considered in this subsection, where the coordinates of the ER are still assumed to be $\left(0,0,0\right)$. Similar to the last subsection, it can be proven that the UAVs hover at the fixed locations under the optimal flight path, accordingly, the parameter *t* can be ignored in the following part of this paper. The positions of the two UAVs are set as $\left(X_{1},Y_{1},H\right)$ and $\left(X_{2},Y_{2},H\right)$, respectively. Without loss of generality, we set the ordinate of the UAV 2 as $Y_{2}=0\;$. Consequently, when K=1,M=2, the sum-energy maximization problem can be simplified to:

(16) $$\begin{array}{c} \max_{X_{1},X_{2},Y_{1}}\;\;\eta \beta_{0}P\left(\frac{1}{{X_{1}}^{2}+Y_{1}^{2}+H^{2}}+\frac{1}{{X_{2}}^{2}+H^{2}}\right)\;\\ \;\;\;\;\;s.t.\;\;\;\;\;\;\;\;\;\;{\left(X_{1}-X_{2}\right)}^{2}+Y_{1}^{2}\geq \Delta^{2}. \end{array}$$

By observing the optimization problem (16), the following conclusion can be drawn.

**Proposition** **1.** *The equation ${\left(X_{1}-X_{2}\right)}^{2}+Y_{1}^{2}=\Delta^{2}$ always holds under the optimal flight path.*

**Proof.**  Please refer to Appendix A

Based on the Proposition 1, the optimization problem (16) can be rewritten as:

(17) $$\begin{array}{c} \max_{X_{1},X_{2},Y_{1}}\;\;\eta \beta_{0}P\left(\frac{1}{{X_{1}}^{2}+Y_{1}^{2}+H^{2}}+\frac{1}{{X_{2}}^{2}+H^{2}}\right)\;\\ \;\;\;\;\;s.t.\;\;\;\;\;\;\;\;\;{\left(X_{1}-X_{2}\right)}^{2}+Y_{1}^{2}=\Delta^{2}. \end{array}$$

□

The Lagrange multiplier method [45] is adopted to solve (17), whose Lagrangian function can be expressed as:

(18) $$L\left(X_{1},X_{2},Y_{1}\right)=\frac{1}{{X_{1}}^{2}+Y_{1}^{2}+H^{2}}+\frac{1}{{X_{2}}^{2}+H^{2}}+\lambda \left({\left(X_{1}-X_{2}\right)}^{2}+Y_{1}^{2}-\Delta^{2}\right),$$

where λ≠0 is the Lagrangian multiplier. The first-order partial derivatives for all variables can be obtained as follows:

(19) $$\frac{\partial L}{\partial X_{1}}=-\frac{2X_{1}}{{\left({X_{1}}^{2}+Y_{1}^{2}+H^{2}\right)}^{2}}+2\lambda \left(X_{1}-X_{2}\right)=0,$$

(20) $$\frac{\partial L}{\partial X_{2}}=-\frac{2X_{2}}{{\left({X_{2}}^{2}+H^{2}\right)}^{2}}-2\lambda \left(X_{1}-X_{2}\right)=0,$$

(21) $$\frac{\partial L}{\partial Y_{1}}=-\frac{2Y_{1}}{{\left({X_{1}}^{2}+Y_{1}^{2}+H^{2}\right)}^{2}}+2\lambda Y_{1}=0,$$

(22) $$\frac{\partial L}{\partial \lambda }={\left(X_{1}-X_{2}\right)}^{2}+Y_{1}^{2}-\Delta^{2}=0.$$

The optimal flight path of the two UAVs can be obtained by separately considering the following two cases.

***Case 1***: When $Y_{1}=0$, based on (19) and (20), we have:

(23) $$\frac{X_{1}}{{\left({X_{1}}^{2}+H^{2}\right)}^{2}}+\frac{X_{2}}{{\left({X_{2}}^{2}+H^{2}\right)}^{2}}=0,$$

with which $X_{1}=-X_{2}$ can be achieved. Without loss of generality, let us assume $X_{1}>X_{2}$, and combining this with (22), we can obtain X1=ΔΔ22, X2=−ΔΔ22. Therefore, the optimal flight path of these two UAVs is to hover over the coordinates of ΔΔ22,0,H and −Δ−Δ22,0,H during the period $\left[0,T\right]$. Additionally, the optimal total energy in this case is:

(24) $$E_{K=1,M=2}^{Y_{1}=0}=\frac{2\eta \beta_{0}PT}{\frac{\Delta^{2}}{4}+H^{2}}.$$

***Case 2***: When $Y_{1}\neq 0$, if $X_{1}=X_{2}$, then $X_{1}=X_{2}=0$ can be obtained from (19) and (20), and $Y_{1}=\pm \Delta$ can be obtained by combining (22). If $X_{1}\neq X_{2}$, it can be obtained from (19) and (21) that:

(25) $${\left({X_{1}}^{2}+Y_{1}^{2}+H^{2}\right)}^{2}=\frac{1}{\lambda }=\frac{X_{1}}{\lambda \left(X_{1}-X_{2}\right)},$$

from which $X_{2}=0$ can be easily obtained. Substituting $X_{2}=0$ into (20) obtains $X_{1}=0$, which shows that the case of $Y_{1}\neq 0$ and $X_{1}\neq X_{2}$ is not true. To sum up, when $Y_{1}\neq 0$, we have $X_{1}=X_{2}=0$ and $Y_{1}=\pm \Delta$. At this moment, the total energy harvested by the ER is:

(26) $$E_{K=1,M=2}^{Y_{1}\neq 0}=\eta \beta_{0}PT\left(\frac{1}{\Delta^{2}+H^{2}}+\frac{1}{H^{2}}\right).$$

Next, we compare the total energy $E_{K=1,M=2}^{Y_{1}=0}$ and $E_{K=1,M=2}^{Y_{1}\neq 0}$ collected by the ER when $Y_{1}=0$ and $Y_{1}\neq 0$, respectively. The difference between them is denoted as τ, which can be calculated as follows:

(27) $$\begin{array}{c} \tau =E_{K=1,M=2}^{Y_{1}=0}-E_{K=1,M=2}^{Y_{1}\neq 0}\\ =\frac{2\eta \beta_{0}PT}{\frac{\Delta^{2}}{4}+H^{2}}-\eta \beta_{0}PT\left(\frac{1}{\Delta^{2}+H^{2}}+\frac{1}{H^{2}}\right)\\ =\eta \beta_{0}PT\left(\frac{2}{\frac{\Delta^{2}}{4}+H^{2}}-\frac{1}{\Delta^{2}+H^{2}}-\frac{1}{H^{2}}\right)\\ =\eta \beta_{0}PT\frac{\Delta^{2}\left(\sqrt{2}H+\Delta \right)\left(\sqrt{2}H-\Delta \right)}{\left(\Delta^{2}+4H^{2}\right)\left(\Delta^{2}+H^{2}\right)H^{2}}. \end{array}$$

Obviously, the plus or minus of τ is determined by the polynomial $\left(\sqrt{2}H-\Delta \right)$. When $\Delta >\sqrt{2}H$, τ<0; when $\Delta \leq \sqrt{2}H$, τ≥0.

To sum it up, the optimal path planning results of UAVs in the scenario with K=1, M=2 are summarized in Proposition 2.

**Proposition** **2.** *In a two-UAV-enabled, one-ER WPT system, both UAVs hover at the fixed locations under the optimal path planning, where the total energy received by the ER is:*

(28) $$E_{K=1,M=2}=\max\left\{\frac{2\eta \beta_{0}PT}{\frac{\Delta^{2}}{4}+H^{2}},\eta \beta_{0}PT\left(\frac{1}{\Delta^{2}+H^{2}}+\frac{1}{H^{2}}\right)\right\}.$$

**Proof.**  The optimal hovering positions of the two UAVs are given as follows:

When $\Delta \leq \sqrt{2}H$, namely, the safe distance between UAVs is less than or equal to $\sqrt{2}$ times of the flight altitude, the two UAVs should hover at the coordinates of ΔΔ22,0,H and −Δ−Δ22,0,H, respectively. On the other hand, when $\Delta >\sqrt{2}H$, the two UAVs hover at the coordinates of $\left(0,0,H\right)$ and $\left(0,\pm \Delta ,H\right)$, respectively.

To be more specific, when $\Delta \leq \sqrt{2}H$, the two UAVs hover symmetrically on either side of the ER. When $\Delta >\sqrt{2}H$, one UAV hovers over the ER, while the other UAV hovers on the side of the ER. At this moment, the relative locations of the two UAVs are no longer symmetrical. To draw more insights, we consider an extreme case, namely, when the safe distance of UAVs tends to infinity, i.e., Δ→∞. Naturally, we have $\Delta >\sqrt{2}H$. If we let two UAVs hover on either side of the ER, respectively, then the distance H2+Δ2Δ244 between the UAV and ER also tends to infinity, so ER receives no energy. However, if one UAV hovers directly above the ER while the other hovers on the side of the ER, the ER will receive the energy transferred by the UAV that hovers directly above it, and the total received energy is QΔ→∞=β0PTβ0PTH2H2.

**Figure 2:**
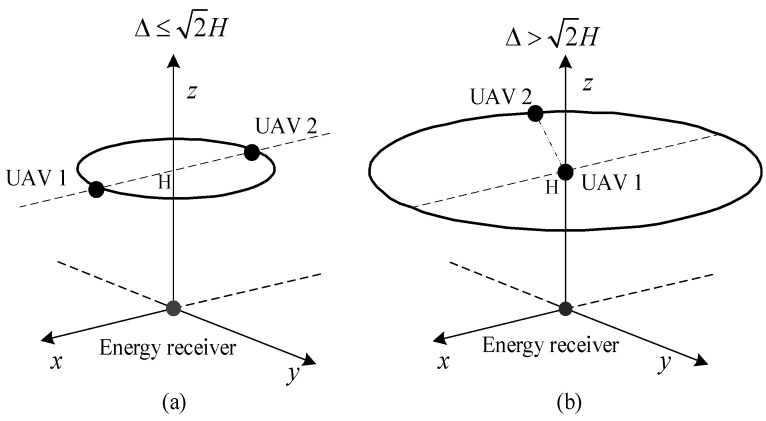
The optimal trajectory of UAVs in the scenario of *K* = 1 and *M* = 2. (**a**): the locations of the UAV when the safe distance $\Delta \leq \sqrt{2}H$; (**b**): the locations of the UAV when the safe distance $\Delta >\sqrt{2}H$.

For illustration, Figure 2 shows the optimal path planning for the UAVs in the special case with K=1, M=2. For ease of analysis, we assume that the ordinate of the UAV 2 as $Y_{2}=0$, while in fact, when $\Delta \leq \sqrt{2}H$, $Y_{2}$ can take any value within −Δ−Δ22,ΔΔ22. Assume the angle between UAV 1 and the *X*-axis to be θ; obviously, $\theta \in \left[0,2\pi \right]$. Consequently, the position of UAV 1 can be written as ΔΔ22·cosθ,ΔΔ22·sinθ,Hθ∈0,π, while UAV 2 is −Δ−Δ22·cosθ,−Δ−Δ22·sinθ,Hθ∈0,π. As long as UAV 1 and UAV 2 are in the circle $\left\{\left.\left(x,y,z\right)\right|x^{2}+y^{2}=\Delta^{2}/4,z=H\right\}$ and symmetric about point (0, 0, H), they can stay static or move at an instantaneous speed not exceeding *V*. When $\Delta >\sqrt{2}H$, UAV 1 hovers at $\left(0,0,H\right)$ while UAV 2 hovers at circle $\left\{\left.\left(x,y,z\right)\right|x^{2}+y^{2}=\Delta^{2},z=H\right\}$, staying static or moving at an instantaneous speed not exceeding *V*. □

## 4. Multiple-UAV-Enabled WPT System with M ≥ 3

According to the analysis in Section 3, the following two useful conclusions can be drawn:

- When $\Delta \leq \sqrt{2}H$, the locations of the UAVs are symmetric, about $\left(0,0,H\right)$. However, if $\Delta \ll \sqrt{2}H$, the safe distance constraint is negligible compared to the flight altitude. In this case, it is obvious that there is a symmetric relationship between any two UAVs in the optimization problem (7), which means that under the optimal path planning, the positions between any two UAVs can be exchanged without changing the relative positions of the UAVs and the ER.
- If one of the UAVs is deployed at a fixed location, the optimal trajectory design for other UAVs is independent of time *t*.

Obviously, when M>2, these two conclusions can be extended with the assumption that $\Delta =\sqrt{2}H$. In this section, the optimal trajectory design scheme of UAVs for the general case of M>2 is considered.

### 4.1. Special Case with M = 3 UAVs

**Figure 3:**
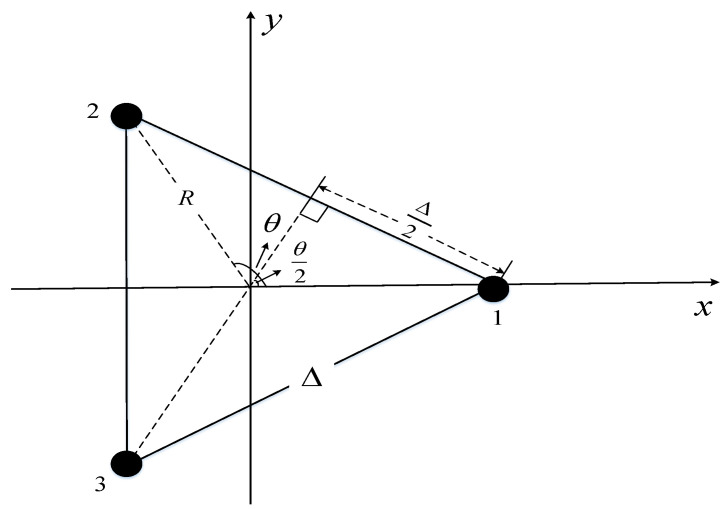
The optimal trajectory of UAVs in the scenario of *K* = 1 and *M* = 3.

The above two conclusions still hold in the three-UAV-enabled WPT system. The relative positions of the *M* UAVs in the optimal path should be a regular polygon with the distance between them being a safe distance Δ. In other words, the relative position of the *M* UAVs is a regular *M*-sided polygon for which length of each side is Δ. For the purpose of exposition, taking the center of gravity of the regular polygon as the origin of the 2D rectangular coordinate system, while one angle of the regular polygon is divided equally by the *X*-axis. Consequently, we have θ=2π2πMM in Figure 3, and the distance from the UAV to the origin is:

(29) R=ΔΔ22sinθ2,

while the coordinates of the UAV *m* are:

(30) $$\left(R\cdot \cos\left(\theta \cdot \left(m-1\right)\right),R\cdot sin\left(\theta \cdot \left(m-1\right)\right)\right),m=1,\cdots M.$$

The distance between the UAV *m* and the ER is:

(31) $$\begin{array}{c} d_{1,m}=\sqrt{X_{m}^{2}+Y_{m}^{2}+H^{2}}\\ =\sqrt{R^{2}+H^{2}}. \end{array}$$

Substituting Equations (29) and (31) into (5), the total energy received by the ER from *M* UAVs for the three-UAV-enabled WPT system is:

(32) EK=1,M>2=ηβ0PTMΔ2Δ244sin2ππMM+H2,M=3.

Furthermore, if we substitute M=1 and M=2 into the Formula (32), whose results are consistent with those in Section 3, consequently, Formula (32) applies to all cases where M≤3 (when $\Delta =\sqrt{2}H$). In addition, we consider another extreme case with Δ→0, under which the charging efficiency of *M* UAVs when the power is *P* equals to that of one UAV when the power is MP. Obviously, this is reasonable, but the total energy expression $Q_{K=1,M>2}$ for M=3 also applies to the case for M>3.

### 4.2. UAV-Enabled WPT System for M > 3

**Figure 4:**
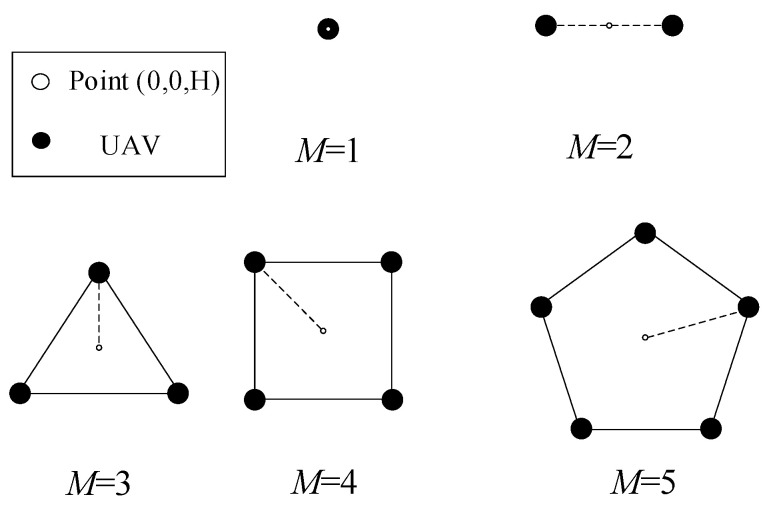
The optimal trajectory of UAVs in the scenario of *K* = 1 and M<6.

As shown in Figure 3, when M=3, all UAVs will hover on the circumference of radius *R* with the safe distance between them. Obviously, when R<Δ, no UAV can hover in the circle or the distance between the UAVs will be less than the safe distance. According to (29), R=Δ can be obtained when M=6, which implies that the total energy expression $Q_{K=1,M>2}$ is applicable to any case for M≤5. For illustration, Figure 4 shows the optimal relative position of UAVs in all cases with M<6. Specifically, when *M* = 5, the radius of the circle that UAVs hover in is R≈0.85Δ, which shows that UAVs can no longer hover within the circle due to the collision avoidance constraint. However, if any UAV hovers outside the circle, the distance between the UAV and the ER will increase, thus reducing the total received energy. Therefore, the optimal trajectory design scheme for M<6 shown in Figure 4 is reasonable.

**Figure 5:**
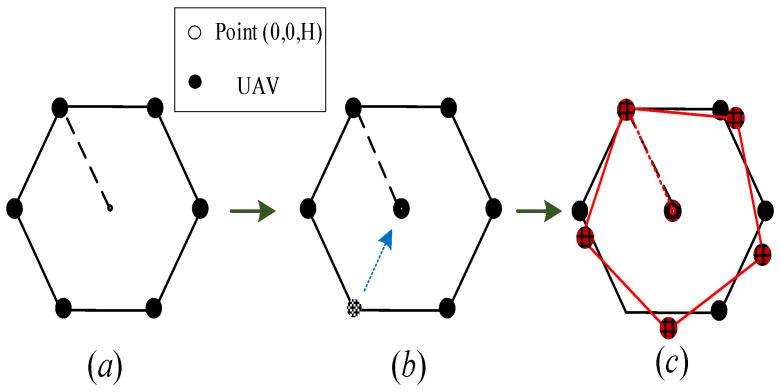
The optimal trajectory of UAVs in the scenario of *K* = 1 and *M* = 6.

Next, the case with M=6 is carefully considered. As shown in Figure 5, the radius of the circle is R=Δ, while the hovering positions in Figure 5a are not any more optimal. In Figure 5b, one of the UAVs is moved to $\left(0,0,H\right)$ and keeps others’ positions unchanged. Under this circumstance, the performance of ER is enhanced due to the closer distance between the ER and the UAVs, while the minimum distance constraints are also satisfied. For the convenience of representation, we assume that the other five unmoved UAVs are evenly distributed on the circumference with $\left(0,0,H\right)$ as the center of the circle and Δ as the radius. As show in Figure 5c by calculation, the minimum distance between the five UAVs is 2sinππ55Δ, which is obviously greater than Δ. At this point, the coordinates of the UAVs can be respectively expressed as:

(33) $$\left\{\begin{array}{c} \left(\Delta \cdot \cos\left(\frac{2\pi }{M-1}\left(m-1\right)\right),\Delta \sin\left(\frac{2\pi }{M-1}\left(m-1\right)\right)\right),m=1,\cdots ,M-1,\\ \left(0,0,H\right),m=M, \end{array}\right.$$

and then the total energy is:

(34) $$E_{K=1,M=2}=\eta \beta_{0}PT\left(\frac{M-1}{\Delta^{2}+H^{2}}+\frac{1}{H^{2}}\right),M=6.$$

When M=7, we just add one more UAV on the circumference based on the case of M=6 and make the UAVs evenly distributed on the circumference. So, Formula (34) also works for the case of M=7.

### 4.3. UAV-Enabled WPT System for K = 2

Suppose that the coordinates of these two ERs are denoted as −Δ−Δ22,0,0 and ΔΔ22,0,0. Under the optimal trajectory design scheme, the UAV hovers at the coordinates of $\left(x,0,H\right)$ based on the analysis conclusions of Section 3 and Section 4. Therefore, the received powers of two ERs, $P_{1}\left(x\right)$ and $P_{2}\left(x\right)$ can be expressed as:

(35) P1(x)=ηβ0P1X−Δ−Δ222+H2,

(36) P2(x)=ηβ0P1X+Δ−Δ222+H2.

At this moment, the total energy harvested by the ER is:

(37) $$E_{K=2,M=1}\left(x\right)=\eta \beta_{0}PT\left(\frac{1}{{\left(x-\frac{\Delta }{2}\right)}^{2}+H^{2}}+\frac{1}{{\left(x+\frac{\Delta }{2}\right)}^{2}+H^{2}}\right).\;$$

Let $f\left(x\right)=E_{K=2,M=1}\left(x\right)$, and then the first-order partial derivatives for *x* can be obtained as:

(38) $$\begin{array}{cc} \; & f^{{}^{\prime }}\left(x\right)=-4\eta \beta_{0}PTx\frac{x^{4}+2\left(\frac{\Delta^{2}}{4}+H^{2}\right)x^{2}-\frac{3\Delta^{4}}{16}+H^{4}-\frac{H^{2}\Delta^{2}}{2}}{{\left(x^{2}+\frac{\Delta^{2}}{4}+H^{2}-\Delta x\right)}^{2}{\left(x^{2}+\frac{\Delta^{2}}{4}+H^{2}+\Delta x\right)}^{2}} \end{array}$$

(39) $$\begin{array}{c} =-4\eta \beta_{0}PT\frac{x\left(x^{2}+A+B\right)\left[x^{2}-\left(-A+B\right)\right]}{{\left(x^{2}+A-\Delta x\right)}^{2}{\left(x^{2}+A+\Delta x\right)}^{2}}, \end{array}$$

where $A=\frac{\Delta^{2}}{4}+H^{2}$ and $B=\sqrt{\frac{\Delta^{4}}{4}+H^{2}\Delta^{2}}$. Furthermore, let $g=\sqrt{B-A}$, and we can obtain the expression of *g* as:

(40)g=Δ44+H2Δ2−(Δ24+H2)(41) <Δ44+H2Δ2+H4−(Δ24+H2)(42) =(Δ22+H2)−(Δ24+H2)(43) =Δ2

- When $\Delta \leq \frac{2H}{\sqrt{3}}$, we can obtain x=0 by calculating the maximization of f(x). The UAV should hover at the coordinate of $\left(0,0,H\right)$ based on the optimal trajectory design scheme. At this moment, the total energy harvested is:
  (44) $$E_{K=2,M=1}=\frac{2\eta \beta_{0}PT}{\frac{\Delta^{2}}{4}+H^{2}}.$$

- When $\Delta >\frac{2H}{\sqrt{3}}$, we can obtain x=g or x=−g by finding the maximization of f(x). The UAV could hover at the coordinate of $\left(g,0,H\right)$ or $\left(-g,0,H\right)$ based on the optimal trajectory design scheme, where $g=\sqrt{\sqrt{\frac{\Delta^{4}}{4}+H^{2}\Delta^{2}}-\left(\frac{\Delta^{2}}{4}+H^{2}\right)}.$ At this moment, the total energy harvested is:
  (45) $$E_{K=2,M=1}=\eta \beta_{0}PT\left(\frac{1}{{\left(g-\frac{\Delta }{2}\right)}^{2}+H^{2}}+\frac{1}{{\left(g+\frac{\Delta }{2}\right)}^{2}+H^{2}}\right).$$

## 5. Simulation Results

In this section, we present numerical results to examine the performance of the proposed algorithms for the path design scheme of UAVs in the single-user model. In particular, the total collected energy of the ER with different parameter settings is provided to validate the effectiveness of the trajectory design proposed in this paper. The specific simulation parameters are shown in Table 1. The transmitted power of one UAV is 40 dBm, and the safe distance between two UAVs is 1m. Unless otherwise specified, the parameters given in the table are used by default. It is assumed that the considered scenario is in an open space with few buildings, and the LoS channel is adopted when the UAVs fly at an altitude of 5 m. For the sake of studying the total collected energy, we measure it by the average received power, which can be calculated by dividing the total collected energy by the UAV’s flight period *T*.

**Figure 6:**
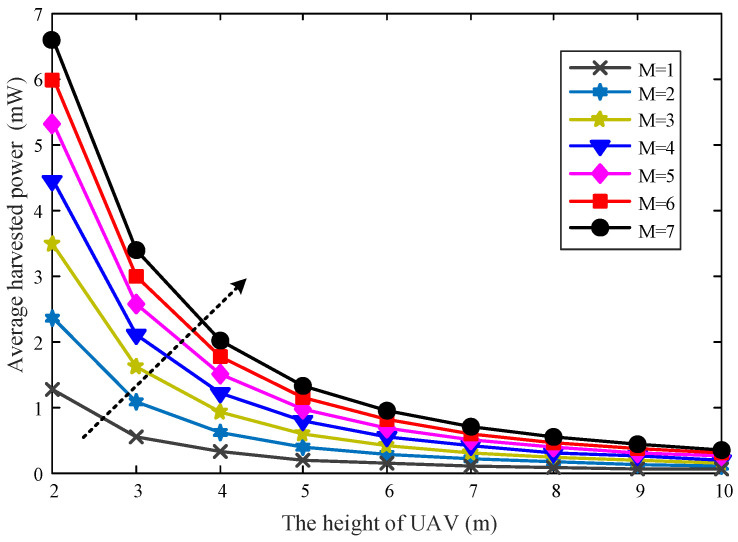
The average received power versus the UAV’s height *H*.

In Figure 6, the relationship between the average received power and the different height of UAV is investigated under the different number of UAVs. It can be seen that the average received power decreases with the increasing height of the UAV because the channel gain decreases as the increasing distance between the UAV and the ER. In addition, the average received power will also increase with the increasing number of UAVs. This is consistent with the previous analysis results in Section 4.

**Figure 7:**
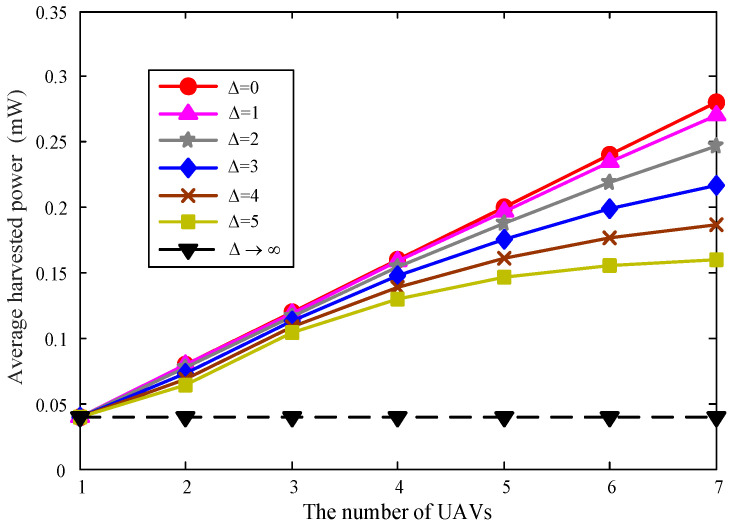
The average received power versus the number of UAVs *M*.

To illustrate the effects on the received power, Figure 7 displays the average received power with respect to the number of UAVs with different safe distances. It is obvious that when the safe distance is Δ=0, the average received power is proportional to the number of UAVs *M*, which validates the analysis in Section 4, and for which the slope is exactly ηβ0Pηβ0PH2H2. As the safe distance increases, the average received power would decrease. This is expected since the fact that the increasing the safe distance must force some UAVs hovering on the circumference to fly farther away, which resuls in the distance between them and the ER increasing. However, when the safe distance approaches infinity, the average received power will not change with the increase of the number of UAVs according to the analysis in Section 4. Specifically, when the safe distance is too large, one UAV hovers directly above the ER while the others have to fly far away because of the limitations of safe distances, and they no longer contribute to the performance of collected energy. At this moment, the average received power can be considered as the average power of a single-UAV-aided WPT system, which is specifically ηβ0Pηβ0PH2H2.

## 6. Conclusions

This paper studied the energy transferred to the ER maximization problem in a multiple UAVs-aided WPT system. We optimized the cooperative UAVs’ trajectories as a new design degree of freedom, subject to the UAV’s maximum flying speed constraints, as well as collision avoidance constraints. First, we considered the basic one/two-UAV scenarios, which indicated that UAVs should hover at the fixed positions for the entire charging period. Based on this solution, we further proposed two useful conclusions to discuss the trajectories of UAVs when the number of UAVs increases from three to seven. The obtained trajectory solutions showed that UAVs should be uniformly deployed in a circle with center point $\left(0,0,H\right)$ and a radius equal to the UAVs’ safe distance. Numerical results were presented to validate the trajectory design algorithm for the multiple-UAV-enabled single-user WPT system.

There are a number of interesting issues not addressed in this paper on account of the space limitation, which we will briefly discuss in the following to motivate future work. Firstly, we suppose that the UAVs-to-ER channels are LoS-dominated; thus, the free-space path loss model is employed in this paper, similar as in prior works [9,10,15,20]. However, obstacles and rich scatters between the UAVs and ER should be considered in certain practical applications such as in mountainous area or forests. How to optimize the UAVs’ trajectories under different loss exponents is a problem worthy of further study. In addition, this paper only considered a single ER served by multiple UAVs, while how to extend the multiple-UAV-enabled single-user WPT to a general multiple-user WPT is another interesting direction.

## Figures and Tables

**Table 1:** Simulation parameters of single-user model.

Parameter	Value
UAV’s transmit power	40 dBm
UAV’s height	5 m
Channel gain	−30 dB
Safe distance	1 m

## Data Availability

Not applicable.

## Appendix A. Proof of Proposition 1

Firstly, we assume that the proposition is not true, namely, ${\left(X_{1}-X_{2}\right)}^{2}+Y_{1}^{2}>\Delta^{2}$.

When $Y_{1}\neq 0$, we can always find ε, which satisfies $0<\varepsilon <\min\left(\left|Y_{1}\right|,\sqrt{{\left(X_{1}-X_{2}\right)}^{2}+Y_{1}^{2}-\Delta^{2}}\right)$. Additionally, we set the ordinate of the UAV 1 as ${Y_{1}}^{\prime }=\pm \sqrt{Y_{1}^{2}-\varepsilon^{2}}$; apparently, the new coordinates $\left(X_{1},{Y_{1}}^{\prime },H\right)$ satisfy the constraint in (16). On the other hand, the optimal value of problem (16) with the new coordinates can be expressed as:

(A1) $$\begin{array}{c} \eta \beta_{0}P\left(\frac{1}{{X_{1}}^{2}+Y_{1}{{}^{\prime }}^{2}+H^{2}}+\frac{1}{{X_{2}}^{2}+H^{2}}\right)\\ =\eta \beta_{0}P\left(\frac{1}{{X_{1}}^{2}+Y_{1}^{2}-\varepsilon^{2}+H^{2}}+\frac{1}{{X_{2}}^{2}+H^{2}}\right)\\ >\eta \beta_{0}P\left(\frac{1}{{X_{1}}^{2}+Y_{1}^{2}+H^{2}}+\frac{1}{{X_{2}}^{2}+H^{2}}\right). \end{array}$$

It is clear that the new coordinates can obtain a higher total received energy, which is inconsistent with the original coordinates being the optimal flight path.

When $Y_{1}=0\;$, without loss of generality, let $X_{1}>X_{2}$, namely, $X_{1}-X_{2}>\Delta$. Assume $X_{1}=X_{2}+\delta$ and δ>Δ. Then, the optimal value is:

(A2) $$\eta \beta_{0}P\left(\frac{1}{{\left(X_{2}+\delta \right)}^{2}+H^{2}}+\frac{1}{{X_{2}}^{2}+H^{2}}\right).$$

Let the abscissa of the UAV 1 be ${X_{1}}^{\prime }=X_{2}+\delta^{\prime }$, where $\Delta <\delta^{\prime }<\delta$. Then, the objective function under the new flight path satisfies the following inequality:

(A3) $$\begin{array}{c} \eta \beta_{0}P\left(\frac{1}{{\left(X_{2}+\delta^{\prime }\right)}^{2}+H^{2}}+\frac{1}{{X_{2}}^{2}+H^{2}}\right)\\ >\eta \beta_{0}P\left(\frac{1}{{\left(X_{2}+\delta \right)}^{2}+H^{2}}+\frac{1}{{X_{2}}^{2}+H^{2}}\right). \end{array}$$

Obviously, the new coordinates can achieve a higher total received energy without violating the constraints. This contradicts the condition that the original coordinates are the optimal flight path.

To sum up, Proposition 1 is proven.
